# Incorporating targeted protein structure in deep learning methods for molecule generation in computational drug design

**DOI:** 10.1039/d5sc05748e

**Published:** 2025-10-20

**Authors:** Lucy Vost, Yael Ziv, Charlotte M. Deane

**Affiliations:** a Department of Statistics, University of Oxford Oxford UK deane@stats.ox.ac.uk; b Centre for Medicines Discovery, Nuffield Department of Medicine, University of Oxford Oxford UK

## Abstract

Traditional drug discovery suffers from high costs and low productivity, with compounds frequently failing due to insufficient efficacy or off-target binding. Structure-based approaches aim to address these challenges by directly incorporating protein target information during molecule design, potentially reducing late-stage failures. In this review, we focus on current deep learning methods for structure-based drug discovery. We discuss the range of approaches used to encode and utilise protein structural information, from early shape-based approaches to more recent co-folding models that predict protein and ligand structures as a single task. We aim to provide insight into how deep learning approaches that incorporate structural information can be used to design molecules with enhanced binding potential while maintaining chemical and physical plausibility and offer suggestions as to the future directions of the field.

## Abstract 2

This review examines current deep learning methods for structure-based drug discovery, detailing how various representations of protein pockets can be integrated to encode structural information and design new molecules.

## Graphical abstract

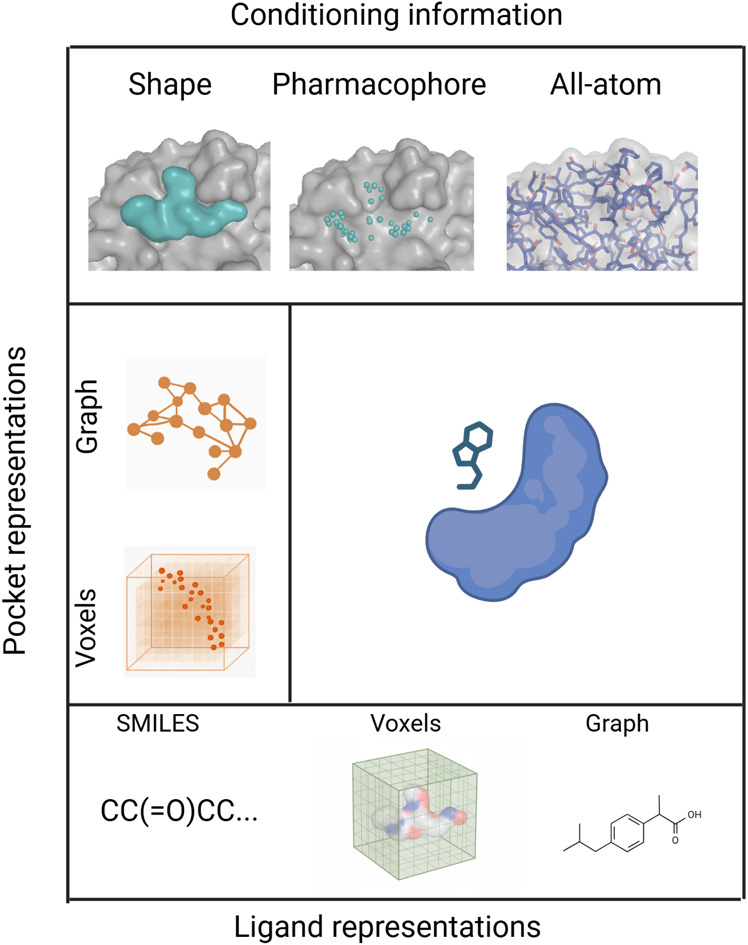

## 1 Introduction

Traditional drug design is costly and time-consuming, with the average expense of bringing a drug from discovery to market estimated at $2.2 billion.^1^ This is largely due to the high failure rate of candidate compounds, meaning that each successful drug must offset the financial burden of numerous unsuccessful attempts.^2^

The reasons for these failures are multifaceted. A 2019 study^3^ reported that in Phase II of clinical trials (where a drug's effectiveness is first tested in patients) a lack of efficacy was the primary cause of failure in over 50% of cases. In Phase III (in which drugs are compared with the best currently available treatment) this figure rose to over 60%. While it might be tempting to assume this simply means the drug does not bind sufficiently strongly to its target, the reality is more complex; failure can also stem from poor “ADME” (Absorption, Distribution, Metabolism, and Excretion) properties. For instance, the drug may be destroyed by stomach acid or be unable to cross the blood–brain barrier. Alternatively, the initial target identification may have been flawed, meaning that modulating the chosen biomolecule does not produce the desired therapeutic effect.^4^

The other primary cause of failure is safety. The same 2019 study^3^ reported that safety concerns consistently accounted for approximately 20–25% of failures across both of these phases. These issues arise from off-target binding, where a drug interacts with unintended biological molecules. Such interactions can lead to adverse reactions.^5^

Accounting for every potential point of failure is practically impossible, not only due to the hugely complex nature of biological systems, but also because negative data from clinical trials is rarely disclosed publicly.^6–8^ Consequently, there is limited systematic data on why and how frequently novel agents fail in late-stage development, making it difficult to learn from failures and reduce them. Given these challenges, a practical strategy to improve the overall success rate is to increase the number of high-quality candidates entering the clinical trial pipeline. The goal is to start with molecules that are already high-affinity, specific binders to the target of interest, thereby improving the odds of success from the outset.

In drug discovery, the design of effective compounds is guided by information about the biomolecular target. This information can be sourced directly from the target's 3D structure in structure-based drug design (SBDD), or indirectly from molecules known to bind to it (known as ligands) in ligand-based drug design (LBDD). Historically, LBDD has been widely employed when a solved structure is unavailable.^9–12^ This remains a common necessity; for example, despite significant advances in structural biology,^13–15^ entire families of pharmacologically vital targets are still largely inaccessible. The most prominent are membrane proteins, which account for over 50% of modern drug targets.^16,17^ Their residence within the cell's lipid membrane creates significant experimental hurdles for structural determination,^18,19^ creating a major discrepancy: while they are a dominant class of drug targets, they constitute only a small fraction of the structures in the PDB.^20,21^ This practical reality ensures that ligand-based design remains an important tool, but it does not negate the method's inherent limitations.

The fundamental limitation of ligand-based methods is that the information they use is secondhand. The difference between the two approaches can be illustrated with an analogy: LBDD is like trying to make a new key by only studying a collection of existing keys for the same lock. One infers the requirements of the lock indirectly from the patterns common to the keys. SBDD, on the other hand, is like being given the blueprint of the lock itself. It allows a key to be engineered by measuring the precise position and nature of each internal tumbler. This direct approach is free from the biases imposed by the original set of keys; for instance, known ligands may possess large chemical substructures that are non-essential for binding or may only probe a limited subset of possible interactions. By avoiding these secondhand inferences, SBDD is inherently more capable of producing truly novel solutions.

While the direct approach of SBDD is powerful, its practical application comes with its own distinct challenge. The feasibility of this approach has greatly increased in recent years as protein structure determination methods—both experimental^22^ and *in silico*^15,23^—have advanced. However, a complete protein structure contains a vast amount of information, much of which is irrelevant to the binding of a specific compound. Therefore, the central challenge in modern SBDD is not just obtaining the structure, but effectively encoding it: distilling the critical structural and chemical features of the binding site from the noise of the surrounding protein. This task of identifying and representing the most significant elements has led to the development of a diverse range of methods.

Machine learning (ML) has emerged as a powerful tool for SBDD, owing to its capacity for pattern recognition and its ability to extract key information from complex data.^24,25^ Early ML approaches built upon physics-based foundations, relying on molecular docking^26–28^ and shape-based ligand generation,^29,30^ but involved manual interventions, such as defining the binding pocket coordinates, selecting specific docking software parameters, or selecting specific interactions for binders to make. As ML models have scaled,^31^ they have become increasingly autonomous, learning to incorporate structural information directly rather than relying on such preprocessed features.^32–34^ This review focuses specifically on generative models. While many machine learning models are designed to predict properties or classify existing data, the purpose of a generative model is to create entirely new data. By training on a large dataset, these models learn the fundamental rules and patterns inherent in the data. For drug design, this means they learn the principles of molecular structure and binding interactions. The model can then use this knowledge to generate novel molecules from scratch, designed to be chemically valid and tailored to a specific protein target.^35^

Nevertheless, a crucial question remains: to what extent do these new approaches genuinely utilise protein information? Evidence for the degree of target structure utilisation is limited, largely due to the absence of standardised, rigorous benchmarks for evaluation. Additional challenges persist, including ensuring the chemical and physical plausibility of generated compounds,^36,37^ achieving generalisability across diverse protein targets,^38^ and accounting for the dynamic nature of protein flexibility in binding interactions.^39^

In this review, we examine why and how protein structure can be integrated into ML methods for three-dimensional ligand generation. Additionally, we discuss future directions and outstanding challenges in the field of structure-based drug design. However, we note that the 3D methods discussed in this review are part of a wider ecosystem of generative machine learning in drug discovery and structural biology. Alongside them, approaches that operate on one-dimensional data are also rapidly advancing. Chemical language models, for example, can learn the ‘grammar’ of chemistry from SMILES strings (strings of ASCII characters representing molecules) to generate novel compounds without structural information,^40^ while other models now design entirely new protein sequences by learning from evolutionary data.^41^ While these text- and sequence-based methods hold promise, they address a different set of challenges. This review will specifically focus on the unique task of incorporating the explicit 3D geometry of a protein target into the generative process, exploring the distinct advancements and hurdles of this structure-based paradigm.

## 2 Overview of drug discovery and development

Before dissecting these machine learning methods, it is important to understand the broader drug discovery pipeline in which they operate. Modern drug discovery is an inherently multi-stage process that integrates biology, chemistry, and clinical research. While strategies vary between therapeutic areas, the canonical pipeline progresses from target identification through hit discovery, lead optimisation, preclinical evaluation, and finally clinical trials.^42,43^

Drug design aims to optimise molecules to achieve a desired therapeutic response by binding to and altering the activity of a biological target, most commonly a protein.^44^ The identification of this target typically relies on genetic or biochemical evidence linking a biomolecule to the disease of interest.^45^ The biomolecule must then be validated, confirming that it is involved in the disease and that modulating it will lead to a therapeutic effect. Once a target is validated, hit identification methods are employed to discover molecules capable of binding to it and perturbing its function. These may include high-throughput screening (HTS), fragment-based drug discovery, or *in silico* screening approaches.^4,5^

Hits are then refined *via* hit-to-lead and lead optimisation campaigns, which iteratively improve properties such as binding affinity, selectivity, solubility, and ADME-T characteristics.^46^ This optimisation typically follows the design–make–test–analyse (DMTA) cycle; the discovery cycle through which molecules are designed, synthesised, and assayed to produce data that in turn are analysed to inform the next iteration.^47^ Preclinical testing further characterises pharmacokinetics and pharmacodynamics while screening for toxicity.^48^ Despite this rigorous process, attrition rates remain high: as per the 2019 mentioned above, fewer than 10% of candidates entering clinical trials ultimately achieve regulatory approval.^3^

The focus of this review is the hit identification stage. Improving efficiency and reliability at this early stage has the potential to reduce attrition downstream, lowering costs and increasing the probability of clinical success.

## 3 Importance of structural insights

A key driver of attrition in drug development is the failure of candidate molecules due to insufficient potency (the amount of drug needed to produce an effect), poor selectivity, or unacceptable toxicity.^49^ Structure-based drug design seeks to help address these challenges by leveraging 3D information about the target itself to rationally guide the design and optimisation of hits.^47,50^

Generative approaches capable of reliably designing molecules with high binding affinity to specific targets would be a huge advance in the field of medicinal chemistry. Their value is twofold. First, molecules tailored to a target structure should reduce (though not eliminate) late-stage failures from lack of efficacy. Second, enlarging the pool of structurally informed candidates increases the chances of identifying compounds with acceptable safety profiles, as highly selective molecules are, by definition, less likely to cause off-target effects.^51^

For computational models to be effective, they must use the structural information available for a target, rather than relying only on indirect measures. Docking provides a rough, physics-based estimate of how well a molecule might bind, and including such scores can help guide models towards more realistic candidates (see Section 4.3). But docking is imperfect: if models optimise only for these scores, they risk producing compounds that appear broadly active but lack true specificity, similar to ‘frequent hitters’ in screening experiments that give false signals across many assays.^52^ On the other hand, ignoring docking altogether and training only on known active molecules can trap models in familiar chemical space, biasing them towards existing scaffolds and limiting the discovery of new ones.

Thus, an essential challenge for SBDD in the generative modelling era is twofold: (i) to develop representations and training strategies that faithfully encode protein–ligand interactions, and (ii) to establish rigorous, standardised benchmarks for evaluating the novelty and specificity of generated compounds. Achieving the right balance between specificity and novelty is critical if computational design is to deliver clinically promising candidates.

## 4 Practicalities of ML in drug design

Implementing ML methods in structure-based drug design involves several practical considerations that shape the approach. The spectrum of human involvement—from expert-guided to fully automated systems—presents tradeoffs in bias management, cost, and molecular plausibility. The starting point for design similarly influences outcomes: *de novo* generation explores broader chemical space, while fragment-based approaches start with small chemical fragments known to bind to the target and then iteratively grow or link them to create a larger, more potent molecule, which can improve synthetic feasibility at the cost of diversity.^53^ Once a strategy is chosen, further complexities arise in implementation, including decisions on protein representation techniques, dataset selection, and evaluation metrics. This section explores these fundamental components that underpin ML-based SBDD strategies.

### 4.1 Incorporating target information

A central challenge in SBDD is how to effectively integrate target-specific structural information into generative models. This must be considered in terms of the granularity of the protein description, the molecular encoding strategy, and the amount of expert preprocessing applied.

Initially, to keep computations tractable, many methods used abstract representations of the protein pocket. Shape-conditioned frameworks such as DESERT and SC-Diffusor, for example, encode the binding site on voxel grids (essentially dividing the 3D space into cubes, analogous to pixels in a 2D image) to capture the coarse geometry required for a good steric fit.^30,54^ To introduce chemical specificity beyond just shape, other approaches imposed pharmacophoric constraints. These are abstract maps of the key interaction points, such as hydrogen-bond donors and acceptors, that a ligand must satisfy to bind effectively. Methods like DEVELOP and STRIFE pre-compute these critical points and use them as a sparse set of anchors to guide a graph-based generator, meaning the protein is represented by a few key constraints rather than its entire dense atomic structure.^55,56^

While abstract representations offered computational efficiency, the pursuit of higher biophysical fidelity and the desire to learn interactions from the ground up led to the adoption of all-atom models, which have since become the dominant paradigm.^32,34,57,58^ The way molecules are encoded for these models has also progressed. Early work relied on voxel-based encodings to generate continuous atomic density maps—a blurry “cloud” of where atoms should be—which required a separate atom-fitting step to produce a discrete molecule.^59^ This limitation was removed as advances in geometric deep learning enabled direct 3D graph representations, where molecules are built as networks of atoms (nodes) and bonds (edges) with precise coordinates and types. This shift to graphs was a pivotal advance, as they not only represent molecules more naturally but also provide a more robust framework for incorporating equivariance. A model is equivariant if a transformation to its input (*e.g.*, rotating the pocket) results in an equivalent transformation to its output (*e.g.*, the generated atoms rotate accordingly). E(3)-Equivariant graph neural networks, as used in Pocket2Mol and related work,^34,60^ guarantee this crucial physical property and have become the standard for atomistic SBDD.

Despite this increased realism, recent systematic benchmarks point to lingering biophysical shortcomings. The PoseCheck benchmark, for instance, showed that seven state-of-the-art generators rarely reproduce the hydrogen-bond networks observed in real crystal structures; for many models, the most common number of interacting donors and acceptors in generated ligands was zero.^61^ In response, the community has begun developing hybrid methods that synthesise detailed atomistic backbones with optional expert guidance. MolSnapper and DiffSBDD, for instance, explicitly condition generation on pharmacophoric points or the pocket geometry, coupling the expressive power of all-atom graphs with user-defined constraints to produce more viable candidates.^33,62^

### 4.2 Datasets

The development of SBDD models is intrinsically linked to the data on which they are trained, and the landscape of available datasets reflects the field's core challenges. The scarcity of high-quality, unbiased experimental data has driven the creation of diverse resources, each with its own strengths and inherent limitations.

The foundation of SBDD is high-resolution experimental data, primarily sourced from the *PDB*, the foundational repository for 3D structural data of biological macromolecules, containing over 230 000 experimentally determined structures as of 2025.^63^

From this vast resource, more focused subsets have been curated to train and validate models. These include the *PDBbind* dataset,^64^ which provides experimentally measured binding affinities for ∼20 000 protein–ligand complexes; *Binding MOAD* containing ∼40 000 protein–ligand complexes with binding data, curated to ensure biological relevance and structural diversity;^65^*sc-PDB*,^66^ a collection of ∼16 000 high-quality binding sites curated from high-resolution X-ray data from the PDB; and *BioLiP*,^67^ which combines ∼200 000 structures with biological insights and annotations mined from literature and other specific databases.

However, the limited volume of experimentally determined structures of protein–ligand complexes, coupled with literature that tends to over-report analogues and binding compounds,^68^ can lead to high similarity between training and testing datasets. Such similarity, along with inherent biases, may cause models to make predictions based on incorrect associations from the training data, frequently resulting in failure when faced with novel data. Durant *et al.*^69^ highlighted that models often learn these biases instead of the true biophysical principles underlying ligand–protein interactions.

To supplement the limited volume of experimental structures, the field also makes use of computationally generated datasets, particularly for training large-scale models and for benchmarking. The *CrossDocked* dataset,^70^ for instance, provides ∼22 million synthetic protein–ligand poses generated through cross-docking, typically filtered to ∼170 000 high-quality poses,^34,71^ to dramatically increase the scale of available training data. The *DUD-E* dataset (Database of Useful Decoys: Enhanced)^72^ provides ∼1.4 million computationally generated ‘decoys’ across 102 targets, designed to help models learn to distinguish true binders from non-binders. More recently, the *AlphaFold DB*^15^ has provided >200 million predicted protein structures, with AlphaFill^73^ adding transplanted ligands and cofactors to ∼1.3 million of these structures.

While essential for building large-scale models, this reliance on synthetic data carries a significant risk: models may learn the artifacts of the docking and generation protocols themselves, rather than the underlying physics of binding.^61^

The most recent class comprises modern hybrid and benchmarking datasets. These platforms aim to provide the best of both worlds by enhancing high-quality experimental data with advanced computational methods. For example, *MISATO*^74^ combines quantum mechanical properties and molecular dynamics simulations for ∼20 000 experimental protein–ligand complexes, including refined structures and explicit water simulations, while *PLINDER* – The Protein–Ligand INteractions Dataset and Evaluation Resource^75^ provides 449 383 protein–ligand systems each with over 500 annotations, similarity metrics at protein, pocket, interaction and ligand levels, and paired unbound (apo) and AlphaFold2-predicted structures, and curated train/validation/test splits for rigorous benchmarking.

Finally, a distinct set of complementary resources is the vast ligand-only databases. As they lack protein structures, they are not used to train the final structure-based component of a model. Instead, their value lies in a common training strategy where a generative model is first pre-trained on a massive and diverse set of molecules to learn the fundamental rules of chemistry and drug-likeness. Datasets used for this purpose include ZINC,^76^ MOSES,^77^ QM9,^78^ GEOM-Drugs^79^ or ChEMBL.^80^ After this pre-training step, the model is then fine-tuned (further trained with a smaller, more specific dataset) on a smaller, target-specific set of structures such as the SARS-CoV2 Main Protease (Mpro). Comparing these models presents a challenge, as evaluations typically involve comparing generated molecules solely against known ligands, rather than benchmarking against other models.

Given these diverse datasets and their inherent limitations, several challenges persist. To mitigate the issue of models learning biases,^69^ it is crucial to carefully split the data, ensuring as little overlap as possible between the test set and the training set at both the molecule and protein target level.

### 4.3 Evaluation metrics

The evaluation metrics used to benchmark SBDD methods can be broadly divided into two categories: those that assess the quality of molecules and those that assess the quality of the molecule's pose.

#### 4.3.1 Assessing the quality of molecules

Assessing the quality of molecules tends to involve evaluating the 2D graph representation of molecules, focusing on several key physicochemical properties. Table 1 summarises the commonly used metrics.

**Table 1:** Metrics for assessing the quality of generated molecules

Metric	Description
Validity	Proportion of generated outputs that represent chemically correct molecules. Checked using cheminformatics libraries (*e.g.*, RDKit^81^) to ensure valency and atomic connectivity rules are satisfied
	
Quantitative Estimate of Drug-likeness (QED)	A score between 0 and 1 estimating drug-likeness by combining eight molecular properties: molecular weight, octanol–water partition coefficient (LogP), number of hydrogen bond donors (HBDs), number of hydrogen bond acceptors (HBAs), molecular polar surface area (PSA), number of rotatable bonds (ROTBs), number of aromatic rings (AROMs), and number of structural alerts (ALERTS)^82^
	
Synthetic Accessibility (SA)	Measures ease of synthesis. Computed *via* rule-based methods analysing molecular complexity (*e.g.*, ring strain, rare functional groups) or retrosynthesis-based planning of synthetic routes^83^
	
LogP	Octanol–water partition coefficient, reflecting lipophilicity. Indicates distribution between aqueous and lipid phases. Optimal drug-like range: −0.4 to 5.6 (ref. 84)
	
Lipinski's rule of five	Heuristic for drug-likeness: molecular weight < 500 Da, LogP < 5, HBD < 5, and HBA < 10 (ref. 85 and 86)
	
Diversity	Structural variety among generated molecules, often quantified as average pairwise Tanimoto dissimilarity between molecular fingerprints.^87^ Higher values imply greater chemical diversity
	
Uniqueness	Proportion of distinct molecules generated, computed as unique molecules divided by total molecules. Reflects ability to avoid duplicates
	
Novelty	Proportion of generated molecules absent from the training set. Measures exploration of new chemical space beyond memorised examples

#### 4.3.2 Assessing the quality of poses

Evaluating the quality of generated binding poses often relies on *molecular docking*, a computational technique that predicts the preferred orientation and conformation of a ligand within a protein's binding site, which can be used to estimate binding affinity. The primary metric used is the *docking score*, a numerical value calculated by tools like Vina^88^ or Smina^89^ that estimates this binding affinity. It indicates how well a ligand fits within the binding pocket, where lower scores typically signify stronger, more favourable interactions. Larger molecules tend to receive more favourable (*i.e.*, lower) docking scores simply due to their size,^90^ unless specific penalties for unfavourable interactions are applied. This bias can be mitigated by using a *ligand efficiency* score,^91^ which normalises the docking score by the number of atoms in the molecule. In addition to reporting docking scores, researchers often report the percentage of generated molecules that exhibit a better binding affinity than a reference molecule.

A common method for evaluating machine-generated molecules is redocking. This involves taking the newly generated ligand, removing it from the protein pocket, and then using a conventional docking program to place it back in. This process can be useful for producing a physically refined structure, as the docking algorithm may resolve issues like internal strain or unfavourable atomic clashes that were been present in the initial, raw output. However, this apparent benefit is also a significant drawback. As highlighted by Harris *et al.*,^61^ using redocking to automatically correct these flaws masks the generative model's weaknesses. A model that consistently produces physically unrealistic structures could be judged favourably if evaluated solely on its redocked outputs, as the fundamental failures in its generation process are concealed.

More fundamentally, for structure-based drug design, this approach misinterprets the primary goal of in-pocket generation. The objective is not merely to generate a viable new molecule, but to generate a molecule in a specific pose that establishes a favourable interaction with the target: the molecular structure and its binding pose being highly intertwined predictions. To truly learn the principles of intermolecular binding, a model must understand not only what to build, but also where to place it. As a full redocking can completely move the molecule from its original pose, it prevents any assessment of whether the generative model has actually learned the geometric and chemical rules that govern binding.

A more direct and less disruptive method of evaluation is local optimisation. This approach is gentler because it refines the existing pose rather than discarding it. One strategy is energy minimisation, where small, iterative adjustments are made to the generated pose within a rigid protein pocket to find a more stable, lower-energy state. This is guided by a physics-based force field (*e.g.*, UFF or MMFF94). Another related technique is to use the local optimisation function available in docking software (*e.g.*, Vina/smina), which uses the program's own scoring function to relax the pose.

Crucially, both of these optimisation techniques respect the model's original spatial prediction. They directly test the local stability and physical plausibility of the generated pose, providing a much more faithful evaluation of the SBDD model's true capabilities.

#### 4.3.3 Integrated metrics

##### 4.3.3.1 Shape and color similarity score

Shape and color similarity score evaluates the 3D molecular similarity between generated molecules and a reference molecule, by volumetric comparison and pharmacophoric feature overlap, as detailed in ref. 55. This metric uses two RDKit^81^ functions, based on the methods described in Putta *et al.*^92^ and Landrum *et al.*^93^

##### 4.3.3.2 Physicochemical plausibility

Physicochemical plausibility is evaluated by tools like PoseBusters,^36^ which examines chemical and geometric consistency, including checking for potential steric clashes.

These fundamental components (how a protein is represented, the data it is trained on, and the metrics used for evaluation) define the landscape of modern SBDD. They create distinct families of methods, each with its own strengths and weaknesses, which we will now explore in detail.

## 5 Current progress

**Fig. 1:**
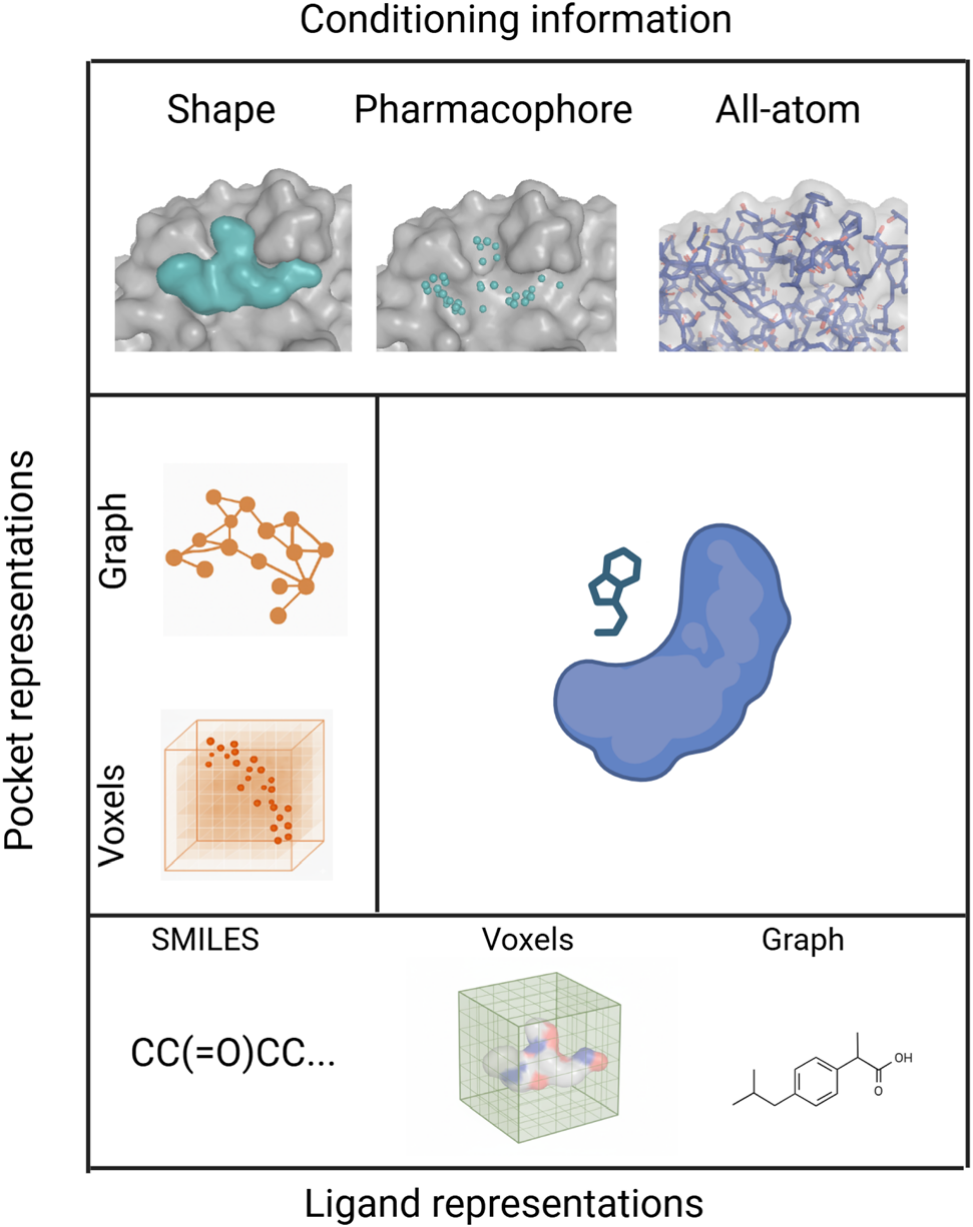
Overview of protein pocket and ligand representations in generative SBDD. The top panel illustrates different levels of *conditioning information* used to describe protein pockets, ranging from simplified shape abstractions to pharmacophore features and detailed All-atom representations. The left axis highlights two main forms of *pocket representations*: graph-based and voxel-based encodings. The bottom panel shows common *ligand representations*, including SMILES strings, voxelised 3D densities, and molecular graphs. Together, these dimensions provide a framework for organising generative methods according to how proteins and ligands are represented. Created with https://www.BioRender.com/jxr55ef.

In this section, we organise existing generative approaches according to how proteins are represented in the generative process (Fig. 1). Along the left axis, methods are first separated into grid-based and graph-based frameworks, reflecting the representation of the protein binding pocket. These pocket encodings can vary in granularity, ranging from simplified shape-based abstractions, through intermediate pharmacophore-level features, up to detailed all-atom representations (top axis). Along the bottom axis, ligand representations are shown, including SMILES strings, voxelised 3D densities, and molecular graphs. This layout provides a systematic view of the design space for generative models in SBDD, highlighting how different combinations of protein and ligand representations define distinct methodological families. Together, these axes define how we group and discuss the methods in this review.

### 5.1 Grid-based approaches

Grid-based methods emerged early in ML-driven SBDD by discretising the continuous three-dimensional space surrounding protein binding sites into regular voxels. This spatial context (including electrostatic fields, hydrophobic regions, and steric constraints) plays a crucial role in determining binding affinity and selectivity. By framing molecular generation as a 3D spatial problem, these approaches capture the geometric complementarity between ligands and their targets. This section reviews recent grid-based methods for SBDD, grouped by the level of protein information encoded in the voxel grid—while noting that ligands may be represented using alternative modalities.

#### 5.1.1 Shape-based

Pocket shape-conditioned grid-based methods discretise the binding site environment into regular voxels, encoding geometric and physicochemical properties—including volume, surface curvature, and electrostatic fields—directly within the 3D grid representation.

An example of a method of this type is DESERT,^30^ in which the authors use ZINC^94^ to train an encoder-decoder network which learns to process voxelised shapes and generate 3D molecules fitting within the specified shape. To address the model's lack of equivariance, the authors introduce random rotations and translations during training, similar to the approach taken by the authors of liGAN^59^ (see Grid-based approaches, All atom).

The authors evaluate DESERT's performance across 12 proteins, finetuning the pocket-unconditional model using available bound data for each protein and optimising generated structures with Vina's local minimisation module. Compared to liGAN^59^ and 3DSBDD,^71^ DESERT shows an improvement in the median Vina score of the top 100 molecules. Moreover, DESERT achieves a higher success rate, with 61.1% of molecules surpassing threshold QED, SA, and Vina score values, compared to liGAN (0.4%) and 3DSBDD (13.6%).

#### 5.1.2 Pharmacophore-based

Pharmacophore-conditioned grid-based approaches embed essential binding features as spatial constraints within voxelised 3D grids. Unlike their graph-based counterparts, these methods exploit the regular grid topology to explicitly map pharmacophoric patterns onto discrete spatial locations, enabling systematic exploration of chemically relevant regions through voxel-based molecular assembly.

An example of a grid-based pharmacophore-conditional method is DEVELOP,^55^ which integrates voxelised 3D pharmacophores (extracted from known binders or provided by the user) with a Convolutional Neural Network (CNN), an architecture adept at processing grid-like data, such as images or the voxelised molecular representations used in this field. It employs this information for linker design or scaffold decoration, converting structures and pharmacophores into graph and voxel grid representations. These are encoded by Graph Neural Network (GNN) and CNN encoders—where the GNN is specifically designed to process molecular graph structures— then decoded into 2D molecule graphs by a GNN-based decoder, following Liu *et al.*'s framework.^95^ The authors evaluate DEVELOP using a shape and color similarity score. They compare generated molecules with ground truth molecules in the PDBBind dataset, and report very high similarity between these: 27.9% of molecules generated present shape and color similarity of over 0.6 with the ground truth ligand. This is considerably higher than other linking methods they compare against, DeLinker^96^ (19.8%) and SyntaLinker^97^—a syntactic pattern recognition approach using deep conditional transformer neural networks—(13.4%).

Another pharmacophore-based graph approach, STRIFE, uses pharmacophoric profiles from known binders with Fragment Hotspot Maps^98^ from protein apostructures to guide fragment elaboration, offering a fully structure-based approach. The model is a descendant of DEVELOP, and uses the same architecture, first encoding the starting fragment and pharmacophore with graph and voxel grid representations respectively, then decoding into 2D molecule graphs by a GNN-based decoder.^95^

The authors of STRIFE conducted a large-scale docking test to assess the binding affinity of generated molecules. They generate molecules for 101 of the targets included in the CASF-2016 test set,^99^ sampling 250 elaborations for each one. They then employ standardised ligand efficiency—a metric derived from docking scores that reflects the difference between the predicted binding affinities of the ground truth and generated molecules—to demonstrate that elaborations generated by STRIFE on average had higher predicted affinities than the original binders. Nevertheless, as a fragment-based approach it requires explicit knowledge of an active fragment, and thus explores a limited region of chemical space, with authors reporting 37.31% uniqueness and 29.21% novelty.

#### 5.1.3 All-atom

While shape- and pharmacophore-based methods simplify the binding site, all-atom grid-based methods attempt to capture its full complexity. They do this by discretising the entire atomic environment, including atom types and positions across a grid of regular cells. This approach offers the potential for higher precision at the cost of greater computational and representational challenges.

An example of this approach is LiGAN,^59^ the first deep generative model aimed at producing 3D compound structures conditioned on receptor binding sites. The method represents molecules using atomic density grids and uses a conditional Variational Autoencoder (VAE) to learn 3D ligand distributions. A VAE is a type of generative model with an encoder-decoder architecture; the encoder compresses input data into a simplified latent space, and the decoder learns to reconstruct the original data from that representation, which allows for the generation of novel samples. Using data augmentation techniques with random rotations to address equivariance, the authors employed the CrossDocked2020 dataset and introduced two distinct sampling modes: posterior and prior sampling. With posterior sampling, a real protein–ligand complex is encoded into the latent variable parameters before drawing samples. Prior sampling, by contrast, draws latent vectors from a standard normal distribution, thus having no intentional bias towards a specific real ligand. The authors report that 98.5% of molecules generated from posterior sampling were valid, while 90.9% from prior sampling were valid. Additionally, 77.7% of posterior molecules and 99.9% of prior molecules were unique.

Generated molecules were also evaluated through energy minimisation experiments using the Universal Force Field (UFF), with the authors assessing the decrease in energy from the generated pose to the minimised pose. Energy decreased on the order of −10^3^ kcal mol^−1^ and −10^4^ kcal mol^−1^ for posterior and prior molecules, respectively, compared to −10^2^ kcal mol^−1^ for real molecules. Moreover, during UFF minimization, the conformation changed by less than 2 Angstrom in 91.3% of posterior molecules and 81.0% of prior molecules. Finally, 30.8% of posterior molecules and 17.3% of prior molecules had lower minimised Vina energy than the reference molecule.

The authors of LiGAN also carry out a comprehensive analysis of pocket conditionality. In a case study involving shikimate kinase, the authors mutated all residues within a specified cutoff distance from the ligand. These multi-residue mutations, along with some key single-residue mutations, resulted in significant changes in the properties of the generated molecules. This demonstrates that the model generates molecules in a manner conditional on the receptor. While this analysis is primarily qualitative and lacks direct comparison to other methods, it remains the sole work to date that rigorously evaluates pocket conditionality in this way. This study represents a step towards establishing regular benchmarking for pocket conditionality. Assessing this aspect in other drug design contexts^100^ has advanced methodologies and may also serve as a valuable metric in generative design.

Wang *et al.*^101^ were the first to introduce a model that leverages experimental electron density (ED) maps as training data. This approach unlocks previously untapped information, including aspects such as non-covalent interactions (NCI), time-averaged conformational changes, and solvent distribution. The model operates with a Generative Adversarial Network (GAN)—a framework that uses two competing neural networks, a generator and a discriminator, to produce realistic outputs—for ligand ED generation and an ED interpretation module for subsequent molecule generation. Like LiGAN, data augmentation is employed to achieve rotational invariance. The authors evaluate the models performance on three targets, reporting improvements in QED and SA over reference molecules for all three (QED averages of 0.54, 0.40, and 0.55 compared to 0.47, 0.32, and 0.49, respectively, and SA averages of 3.0, 3.2, and 2.9 compared to 3.6, 3.2, and 3). The authors also reported similar performances in docking score for the generated molecules and the ground truth binders as assessed by glide.^102^ Overall, this method represents a novel advancement in using a previously untapped source of data for SBDD. However, the richness of information from ED maps brings about challenges associated with data complexity and noise, which could potentially impact the accuracy of generated structures.

RELATION,^103^ built on a VAE, takes a unique approach by transferring geometric features of protein–ligand complexes to a latent space for generation. This model comprises a 3D convolutional encoder and an LSTM-based captioning decoder, with pharmacophore conditioning and docking-based Bayesian sampling guiding molecule generation. Despite its strengths, similar to LiGAN and the method proposed by Wang *et al.*,^101^ RELATION faces challenges related to non-equivariance, resulting in a limited capacity to generate novel binders.^104^ The authors use the ZINC Clean Lead database, then bound data for two target proteins. They observed improved validity over LiGAN (0.994 *vs.* 0.873), though a direct comparison between the two models is difficult: LiGAN is designed to work on any target, trained on diverse bound data, whereas RELATION is specifically tailored for the examined targets. The authors also reported similar Vina score distributions for the generated molecules and the ground truth binders.

In summary, these recent advancements present diverse perspectives on directly modeling target–ligand interactions. While each model introduces innovative features, they all grapple with shared challenges: the lack of rotational equivariance in CNNs, and limited voxelised resolution resulting in an inability to precisely capture specific modes or intricate patterns in the distribution of atom distances.^105^

### 5.2 Graph-based approaches

Graph-based methods emerged next as a dominant paradigm in SBDD due to their natural representation of molecular structures, where atoms serve as nodes and bonds as edges. As topological encodings matured, the field shifted toward more flexible graph neural networks that explicitly model chemical interactions. These frameworks support rich annotations—atom types, bond orders, partial charges—and can integrate protein context at both the residue and all-atom levels. This section reviews recent graph-based methods for SBDD, grouped according to the level of protein information incorporated.

#### 5.2.1 Shape-based

The simplest graph-based methods utilise only the geometric shape of the protein pocket to guide molecule generation. SQUID exemplifies this approach, using point cloud networks and graph neural networks (GNNs) to encode shape and chemical identity; an approach that addresses equivariance and reduces the memory usage of the model. It comprises a fragment-based generative model based on a variational autoencoder, which sequentially decodes fragments to enhance the validity of generated molecules. Once generation is complete, it further modifies the generated conformers by adjusting acyclic bond distances and fixing acyclic bond angles using heuristic rules. As it is a shape-based model, the model is trained on unbound data; specifically, a subset of the MOSES dataset.^77^ The model is first assessed *via* ablation experiments to see if including equivariance improves the model's performance, and it is found that removing equivariance reduces the percentage of generated molecules that have the desired shape by 33%. To assess performance, the authors employ a shape similarity metric that estimates the likeness of the molecules generated to the desired shape. Rather than using docking scores, they compare SQUID's performance to a baseline that searches the training set (>1 M 3D molecules) for the molecule with the highest property score among those that satisfy a shape similarity threshold to the target—a strategy akin to shape-constrained virtual screening—and find improvements in shape matching across six different targets.

#### 5.2.2 Pharmacophore-based

Building beyond pure geometric constraints, pharmacophore-based methods incorporate specific chemical feature requirements into the generation process. An example of this approach is PGMG^104^ which relies on user-supplied pharmacophores, using a GNN and a transformer decoder to generate molecules. Since pharmacophores and molecules have a many-to-many relationship, PGMG introduces latent variables to model such a relationship to boost the variety of generated molecules. In addition, a transformer structure is employed as the backbone to learn the implicit rules of SMILES strings to map between latent variables and molecules. The authors demonstrate that 83.6% of generated molecules achieve matching scores greater than 0.8 with the given pharmacophore, with 78.6% achieving perfect matching score of 1.0. Random molecules from ChEMBL, only had matching scores centered at 0.466 with only 4.91% achieving perfect scores. When evaluated on 15 protein targets, PGMG produced molecules with comparable docking scores to known active compounds. In direct comparisons with Pocket2Mol (see all-atom section) on two of these targets (AKT1 and CDK2), PGMG achieved superior performance in several key metrics. For AKT1, PGMG attained a 99.2% ratio of available molecules compared to Pocket2Mol's 87.2%, though Pocket2Mol achieved slightly better docking scores (−7.81 *vs.* −7.35). Similarly for CDK2, PGMG reached 98.9% available molecules *versus* Pocket2Mol's 90.2%, with comparable docking scores (−7.48 *vs.* −7.55). However, these comparisons are limited to only two targets. More fundamentally, requiring users to define pharmacophores, whether through visual estimation or by referencing known ligands, reintroduces human bias.^106^

#### 5.2.3 All-atom

All-atom methods represent the most comprehensive approach to SBDD, explicitly modeling every atom in both the protein binding site and generated ligands. Unlike shape-based or pharmacophore-based methods that abstract away atomic details, these approaches leverage the full structural information available from protein–ligand complexes, including precise atomic positions, chemical identities, and potential interaction sites. An example of this approach applied with graph representations is the work by Drotár *et al.*,^107^ who introduced the first supervised method for joint molecular graph and pose generation, using a constrained graph VAE approach. Molecules are represented as graphs with atoms defined by bond lengths and angles, guided by crystallography data. Their method embeds all atoms in a latent space and employs MLPs to predict angles and dihedral angles, with bond distances calculated based on atom and bond types. By integrating experimental ligand–protein data, their method enhances predicted binding affinities by 8% and drug-likeness scores by 10% compared to the baseline approach that generates 2D graphs without pocket information on the SMINA docking benchmark.

Another example is the work of Luo *et al.*^71^ who introduced 3DSBDD, an autoregressive generative model that uses the protein pocket as a conditioning constraint to sample ligands. This model calculates the atom occurrence probability density in 3D space of the binding site. It then employs an auto-regressive sampling algorithm, sampling one atom at each step using Markov Chain Monte Carlo sampling. 3DSBDD infers bonds heuristically from the generated atomic point clouds and uses a point cloud representation for both ligand and protein. Compared to the CNN baseline liGAN^59^ (see Grid-based approaches, All-atom), 3DSBDD improved the QED (0.525 *vs.* 0.371), SA (0.650 *vs.* 0.570), and Vina Score (−6.200 *vs.* −6.100) metrics on the CrossDocked dataset.

Liu *et al.* proposed GraphBP,^60^ representing the protein binding pocket and the partially constructed ligand as a single graph. At each step, a 3D graph neural network uses this evolving graph to predict the next atom's type and 3D coordinates, embedding both geometric structure and chemical interactions. GraphBP creates a local coordinate system for new atom placement, ensuring equivariance, and uses a flow model for atom type and position prediction. This approach enables continuous atom placement, offering greater flexibility compared to methods like 3DSBDD. GraphBP also generates more valid molecules (99.7% compared to 98.5% for liGAN), with better predicted binding affinity, with 27% generated molecules having higher predicted binding affinity than their corresponding reference molecules compared to 15.4% for liGAN.

Peng *et al.*^34^ developed Pocket2Mol, an evolution of 3DSBDD. In Pocket2Mol, bonds are predicted directly during sequential ligand generation. Pocket2Mol's E(3)-equivariant graph neural network architecture respects 3D spatial symmetries and efficiently captures spatial and bonding relationships without the need for Markov Chain Monte Carlo methods, which are typically less efficient. This innovation means Pocket2Mol is the current state-of-the-art for the SA (0.765) and QED (0.563) metrics on the CrossDocked dataset.

In contrast to the above atom-based methods, the FLAG model^108^ selects fragments from a predefined motif vocabulary based on protein structure and iteratively assembles them into a complete ligand. Using a 3D graph neural network, FLAG encodes contextual information, facilitating the selection and combination of motifs for an optimised ligand–target interaction. This approach then generates molecules fragment by fragment, requiring fewer steps and thus offering faster processing. The authors compare FLAG to LiGAN (section Grid-based approaches, All-atom), Pocket2Mol, and GraphBP, and report improved Vina Scores (−7.247 compared to −6.129, −7.113 and −7.012, respectively) and SAs (0.745 compared to 0.612, 0.733 and 0.706). These results are computed after the generated molecules are redocked: a method which the PoseCheck work^61^ highlighted as masking clashes between the ligand and the protein and increasing interactions between them, resulting in inflated docking scores.

Overall, graph-based approaches have emerged as a powerful paradigm in structure-based drug design, offering several key advantages. Their natural representation of molecular topology enables efficient learning from irregular geometries while maintaining permutation invariance—ensuring consistent predictions regardless of atom ordering. From shape-based methods like SQUID to sophisticated all-atom approaches, these models have demonstrated some ability to generate valid, drug-like molecules with promising binding affinities across diverse protein targets.

### 5.3 Diffusion models

Recent research has shifted towards diffusion models^109^ due to their ability to capture both local and global atomic interactions by placing all atoms simultaneously, rather than generating molecules atom by atom in prior autoregressive approaches. This holistic generation allows reasoning over entire molecular structures in one pass and typically enables faster sampling. Diffusion is a generative *method* rather than a new representational class. In principle, diffusion can be applied to different representations, including voxel- and graph-based formulations. In practice, graph-based diffusion models have become the dominant approach in SBDD, and we therefore discuss them in detail within the context of graph-based methods.

#### 5.3.1 Shape-based

Pocket shape-conditioned diffusion-based methods leverage binding site geometry through iterative denoising processes that gradually refine molecular structures from random noise. By conditioning the diffusion process on pocket descriptors—including volume, surface curvature, and electrostatic fields—these approaches guide the reverse diffusion trajectory to generate molecules that naturally complement the binding site architecture through progressive structural refinement.

An example of this approach is ShapeMol,^54^ which uses an equivariant approach, relying on an SE(3)-equivariant diffusion model based on the work of Hoogeboom *et al.*^110^ to generate molecules in a point-cloud specified shape. ShapeMol does not impose adjustments on the generated 3D conformers, enabling it to accept any conformers as input. This increases the uniqueness of molecules made, but combined with the flexibility to use atom-level generation results in lower validity of generated molecules, particularly with a diffusion model known for issues in generating chemically sensible structures.^61^ Following SQUID (Graph-based approaches, shape-based), the authors of ShapeMol use a subset of MOSES as a training dataset, and evaluate performance using a shape similarity metric. They compare themselves to SQUID and find improvements in this metric, though they report slightly worse molecule connectivity (98.8% *vs.* 100%) and QED (0.748 *vs.* 0.766).

#### 5.3.2 Pharmacophore-based

Pharmacophore-conditioned diffusion-based approaches incorporate essential binding features as conditioning signals within the denoising process. Unlike grid-based methods that operate on discrete voxels, these diffusion models use pharmacophoric constraints to bias the continuous sampling trajectory, enabling flexible molecular generation that satisfies key interaction requirements through iterative noise removal.

While previous pharmacophore-conditioned methods generated 1D SMILES strings or 2D molecular graphs and then generate conformers and dock these, MolSnapper^62^ employs a generative diffusion model that integrates 3D pharmacophores and protein structural information to produce 3D ligands. Specifically, it conditions MolDiff,^111^ an E(3)-equivariant neural network, to generate molecules that fit into a binding pocket. Evaluation focused on the physical and chemical viability of the generated molecules. Results on the CrossDocked and Binding MOAD datasets demonstrate MolSnapper's ability to yield twice as many valid molecules as competing methods (MolDiff,^111^ SILVR,^112^ and DiffSBDD^113^) and offers up to a 20% improvement in shape and color similarity to reference ligands, leading to a 30% better retrieval of initial hits over these methods.

#### 5.3.3 All-atom

All-atom conditioned diffusion-based methods condition the denoising process on complete atomic-level representations of the protein binding site. Rather than discretising space into grids, these approaches use the full atomic environment—including precise atom types, positions, and chemical contexts—to guide the continuous diffusion sampling process, enabling detailed modeling of protein–ligand interactions through probabilistic molecular generation.

One such model is DiffSBDD,^113^ a SE(3)-equivariant 3D conditional diffusion model that respects translation, rotation, and permutation symmetries. It represents proteins and molecules as 3D point clouds, using an EGNN architecture to diffuse only atom positions and types, along with a *post hoc* bond order approximation. This method produces relatively diverse ligands, evidenced by a 0.758 Tanimoto dissimilarity among all generated molecules for each pocket, narrowly outperforming Pocket2Mol (0.735), TargetDiff^105^ (0.718) and 3DSBDD (0.742), though it is substantially outperformed by GraphBP (0.844). It also achieves an improved average Vina docking score at −7.333 compared to these methods, which attain −7.058, −7.318, −5.888, and −4.719 respectively. However, it does not improve molecular properties such as QED and SA on the CrossDocked dataset when compared to Pocket2Mol.

TargetDiff,^105^ conceptually similar to DiffSBDD, also represents proteins and molecules as 3D point clouds, diffusing only atom positions and types, utilising a different diffusion formalism for categorical atom types. It shows similar outcomes, primarily improving Vina docking scores (−7.80 after redocking and 58.1% molecules show better binding affinity than the reference molecule, compared to −7.15/48.4% for Pocket2Mol, and −6.33/21.2% for liGAN) without significantly affecting other molecular properties.

DiffBP^32^ introduces a pre-generation network for the ligand's center of mass and atom number, followed by diffusion models and equivariant GNNs for ligand generation. It demonstrates high docking scores, with 40.20% of medium-sized molecules exhibiting improved docking scores over the reference molecule, outperforming 3DSBDD (14.84%), Pocket2Mol (32.53%), and GraphBP (15.30%) on the CrossDocked dataset. The analysis and evaluation distinctly categorise molecules into small, medium, and large, acknowledging that larger molecules typically achieve higher docking scores.

Existing diffusion model-based methods encounter limitations, particularly in bond incorporation, which often results in the creation of unrealistic molecular structures.^111^ DecompDiff^57^ was developed in response to these challenges, aiming to improve molecular generation by adding prior knowledge and explicitly modeling bonds. This model employs data-dependent decomposed priors for SBDD, a strategy that acknowledges the natural decomposition of a ligand molecule into functional regions such as arms and a scaffold. These decomposed priors have led to improvements in affinity-related metrics. However, like other diffusion models, DecompDiff does not exceed the performance of the state-of-the-art autoregressive model Pocket2Mol in terms of QED and SA scores.

Recently, the field has begun shifting from traditional diffusion models toward flow-matching approaches^114^—a closely related class of generative models that offer improved training stability and deterministic sampling without the need for iterative denoising. For example, FLOWR^115^ demonstrated improved PoseBusters validity (86% *vs.* 75% for diffusion-based models) and better Vina docking scores (−6.36 *vs.* −6.06) on a benchmark derived from the PLINDER dataset. Another flow-matching model, FlexSBDD,^116^ incorporates protein flexibility by jointly generating both the ligand and key degrees of freedom in the protein binding site—namely the C_*α*_ coordinates, backbone orientation, and side-chain dihedral angles—to reconstruct full-atom protein structures and better capture induced-fit effects during design.

In a recent paper, Harris *et al.*^61^ found that diffusion-based models tend to produce structures with higher strain energy compared to those in the training dataset. This increased strain might result from the introduction of random noise into coordinate features during most steps of stochastic gradient Langevin dynamics sampling, except the final step. This process complicates the accurate construction of bond angles and other structural details, potentially affecting the realism of the molecules generated.

## 6 Future directions

Having covered the current SBDD methods, we now propose potential future areas and directions.

### 6.1 Assessing specificity

The assessment of binding specificity remains a critical yet underdeveloped aspect of computational ligand design. Among the limited approaches in this domain, the LiGAN methodology^59^ stands out for introducing a quantitative framework to evaluate specificity. The authors implemented a systematic mutation analysis, altering all residues within a defined distance from the binding site as well as specific individual residues of interest. These controlled mutations produced measurable changes in the properties of the generated molecules, providing compelling evidence that the model's outputs are indeed conditional on the receptor's characteristics. Unfortunately, this approach represents an isolated example in the literature, making comparative analysis across methodologies impossible.

The majority of current research relies heavily on docking scores as a proxy for binding quality and specificity. While computationally accessible, these scores are susceptible to optimisation strategies that do not necessarily translate to true binding specificity in biological systems. Another common evaluation approach centers on interactions with known ligands for a target. While this method benefits from target-specific relevance, it inherently constrains exploration to chemical spaces adjacent to established binders. This limitation effectively reduces the potential for novel discovery, approaching the constraints of traditional ligand-based design strategies rather than enabling the broader exploration promised by structure-based approaches.

### 6.2 Generalisation

The challenge of generalisation in SBDD is rooted in the nature of its underlying data. Public protein–ligand datasets were not curated for machine learning; they are the product of decades of structural biology research, leading to a non-uniform sampling of chemical and protein space with inconsistently reported metadata.^117,118^ This creates hidden biases that are well-documented in virtual screening,^69,100,119^ but are harder to diagnose for *de novo* generation. This is in part because the virtual screening tools used to assess generated molecules are, themselves, known to be unreliable on the out-of-distribution examples that are the true test of generalisation. It is therefore impossible to robustly quantify a generative model's performance on novel targets, making it unclear if it is discovering genuinely new interactions or, more likely, simply exploiting biases shared with the evaluation tool.

While long-term solutions involve rectifying the data landscape,^120^ a primary pragmatic strategy in the interim is to simplify the task by constraining the generative process. By building frameworks that allow users to enforce expert knowledge—such as specific chemical rules or pharmacophoric features^56,62^—the model's reliance on learning from biased data is reduced. This approach of ‘informed generation’ grants greater control over the output and provides a path forward while the field awaits more comprehensive datasets.

### 6.3 Protein flexibility

Proteins are dynamic, exhibiting motions and conformational changes that may significantly impact drug interaction and efficacy.^121^ Accurate prediction of these interactions requires a thorough consideration of protein dynamics.^121^

Traditionally, SBDD has heavily relied on static crystal structures. However, a crystal structure represents a single snapshot of a specific protein conformation.^121^ This snapshot is influenced by factors such as the presence or absence of a co-crystallised ligand and may not necessarily capture the stabilised conformation required to achieve the desired downstream bioactivity.^122^

Molecular dynamics (MD) simulations are a widely used method for modeling protein flexibility.^123^ However, it's important to recognise that while MD simulations provide valuable insights, they are computationally demanding and may not always achieve the desired level of accuracy.^124^

Several strategies are being explored to predict structures of multiple protein conformational states. One set of methods rely on manipulating the inputs of AlphaFold 2 (AF2).^15^ By altering the multiple sequence alignment (MSA), researchers aim to deconvolve coevolutionary signals for several conformational states. Strategies like subsampling MSAs to shallower depths have shown promise in increasing the diversity of output models, potentially representing multiple conformations.^125–127^

Another approach is to improve the exploration of contact and distance maps. Contact and distance maps predicted from MSAs contain information about alternative protein conformations. Predicted inter-residue distance distributions sometimes show bimodal characteristics, indicating conformational changes.^128,129^ Hou *et al.*^130^ use the distance maps from AF2 and other tools to construct multiple energy landscapes, identifying low-energy solutions representing potential conformations.

Generative models, such as diffusion models and variational autoencoders, offer a new avenue for conformation prediction tasks.^131,132^ These models can sample distributions of outputs, potentially generating multiple related structures for a given input sequence. For example, the EigenFold^131^ method, a diffusion model, was explored for its ability to sample structures of multiple conformations.

In the context of SBDD, ensuring that these methods can be generalised to a broader range of protein structures and accurately differentiate between viable models and noise remains a significant challenge.

### 6.4 Cofolding methods

Most SBDD models generate ligands against a fixed protein conformation; however, proteins are dynamic entities that undergo significant conformational changes upon ligand binding. Cofolding methods tackle this by jointly predicting protein and ligand structures, allowing both partners to adapt dynamically and capture induced-fit or conformational-selection effects. Cofolding methods aim to jointly predict the three-dimensional structures of interacting biomolecules, such as protein–protein, protein–ligand, or protein–nucleic acid complexes. For protein–ligand interactions, they typically use a known binder—usually provided as a SMILES string—to model the complex, distinguishing them from *de novo* generative methods that design new molecules from scratch.

RosettaFold All-Atom (RFAA)^133^ was one of the first models to handle proteins, nucleic acids, small molecules, and metal ions in the same system, using a transformer architecture with chemical element inputs. AlphaFold3^23^ built on this by adding diffusion-based coordinate generation, which improved accuracy across many types of biomolecular interactions. Since then, several open-source alternatives have appeared. Chai-1^134^ closely follows AF3's transformer-plus-diffusion design but makes the code and weights freely available and easier to train, while Boltz-1^135^ provides similar functionality with faster inference and lower memory requirements. Boltz-2^136^ adds further changes: more efficient training and inference through trunk optimisation, better physical plausibility *via* Boltz-steering, and new conditioning options (method, template, and contact/pocket conditioning) that give users more control. It also includes a dedicated affinity module to predict binding likelihoods and affinities alongside structures. In contrast, NeuralPLexer3 is designed specifically for protein–ligand docking, using physics-informed graph neural networks to model multiple binding poses, affinities, and induced-fit conformational changes.

A recent benchmark by Škrinjar *et al.*,^38^ comprising 2600 high-resolution protein–ligand systems released after these methods' training cutoffs, reveals significant limitations in current cofolding approaches. Their analysis demonstrates that these methods largely memorise ligand poses from training data rather than genuinely predicting novel configurations, severely limiting their utility for *de novo* drug design. While all methods achieve reasonable accuracy in modeling protein structures and binding pockets, ligand pose prediction remains the primary challenge. Despite similar architectures and training paradigms across methods, AlphaFold3 maintains a slight performance edge, potentially due to methodological differences: it uniquely uses templates by default for protein modeling, while training protocols vary significantly—Boltz-1 generates conformers only once during training whereas others regenerate them each epoch, and Chai-1 incorporates ESM embeddings for protein featurisation. Nevertheless, the fundamental finding remains that current cofolding methods are not yet suitable for *de novo* drug design applications.

### 6.5 Other challenges

Using machine learning in SBDD poses a challenge in validating the quality of generated molecules and their binding poses. Recent methods such as PoseBusters^36^ and PoseCheck^61^ have shown that deep learning methods, including those using diffusion models, can produce physically implausible structures.

PoseBusters evaluates chemical and geometric consistency, identifying problems such as incorrect stereochemistry, non-planar aromatic rings, improper bond lengths, and clashes between proteins and ligands. Similarly, PoseCheck notes nonphysical features in machine-generated molecules, such as steric clashes and hydrogen placement issues. For instance, autoregressive methods like 3DSBDD and LiGAN exhibit average steric clashes of 3.79 and 3.40 with the protein, respectively, indicating fewer steric overlaps between the ligand and protein. In contrast, newer diffusion-based approaches, such as TargetDiff and DiffSBDD, report higher mean clash scores of 9.08 and 15.33, respectively, indicating more frequent or severe steric clashes.

Moreover, PoseCheck's evaluation of seven deep learning methods revealed that, in the poses generated, the most frequently observed count of hydrogen bond acceptors and donors in the generated molecules forming interactions was zero. This is a serious deviation from the expected number of interactions. This finding underlines the limitations of traditional 2D-based evaluation metrics, which may fail to capture these critical errors.

To advance SBDD, it is essential to develop benchmarks that not only assess the plausibility of ligands but also the accuracy of binding poses. Such benchmarks must rigorously ensure that binding poses adhere to biophysical requirements essential for effective binding. Improving these evaluation standards is crucial to bridge the gap between theoretical models and their practical clinical applications, ultimately enhancing the discovery of more effective therapeutics.

## 7 Outlook

As the field of generative SBDD continues to evolve, several key challenges and opportunities have emerged that will shape its future application and development.

For practitioners, the current choice between different generative families involves a critical trade-off between precision and exploratory power. Autoregressive models, such as LiGAN and Pocket2Mol, which build molecules atom-by-atom, tend to offer greater control. This often results in generated poses with fewer steric clashes and more plausible interactions, making them well-suited for tasks like lead optimisation where high-quality modifications are paramount. In contrast, diffusion models excel at rapidly generating a large and diverse set of novel chemical ideas. While these models may produce a higher rate of physically implausible structures that need to be filtered, their speed and exploratory capacity make them a powerful tool for hit identification, where the primary goal is to discover new and promising scaffolds.

Beyond these practical choices, a critical area for future research is enhancing the overall quality and reliability of generated molecules. This involves three interconnected challenges: ensuring physical plausibility, improving synthetic accessibility, and establishing standardised benchmarks. Models must produce geometrically and chemically sound structures that adhere to the physical laws of binding, as highlighted by tools like PoseBusters and PoseCheck. Concurrently, generated molecules must be synthetically tractable within the economic constraints of a drug discovery campaign. Finally, the development of rigorous, community-wide benchmarks is essential to allow for fair comparison between methods and to track genuine progress in the field.

A more profound challenge lies in accounting for the dynamic nature of protein targets. Future models must move beyond static structures to capture protein flexibility and the subtle conformational changes induced by ligand binding. Addressing this is key to unlocking more sophisticated pharmacological control, such as allosteric modulation (binding to a secondary site on the protein to influence the main active site from a distance), and accurately predicting a drug's true biological effect.

Looking further ahead, a promising path involves integrating the 3D structure-based methods discussed here with complementary approaches, such as chemical language models. Such hybrid systems could reduce late-stage attrition by tackling multiple failure points at once, leveraging language models to optimise for intrinsic drug-like properties (*e.g.*, ADME/Tox) while structure-based models ensure high-affinity target binding.

In conclusion, refining SBDD models through these various improvements is not just an academic exercise but a necessary evolution for the field. By addressing these issues, we can lay the groundwork for cutting-edge advances in drug design. These advancements hold the promise of delivering more effective therapies to patients faster, ultimately transforming the landscape of modern medicine.

## Author contributions

L. V. and Y. Z. contributed equally to this work. L. V., Y. Z., and C. M. D. conceptualised the scope of the review. L. V. and Y. Z. wrote the original draft and created the visualisations. C. M. D. supervised the project. All authors contributed to the review and editing of the manuscript.

## Conflicts of interest

CMD discloses membership of the Scientific Advisory Board of Fusion Antibodies plc and AI Proteins, and is a founder of Dalton. All other authors declare no conflict of interest.

## Data Availability

No primary research results, software or code have been included and no new data were generated or analysed as part of this review.
